# Treatment of broad-based intracranial aneurysms with the LVIS EVO stent: a retrospective observational study at two centers with short- and medium-term follow-up

**DOI:** 10.1038/s41598-023-34270-0

**Published:** 2023-05-04

**Authors:** Christoph J. Maurer, Ansgar Berlis, Volker Maus, Lars Behrens, Werner Weber, Sebastian Fischer

**Affiliations:** 1 Department of Diagnostic and Interventional Radiology and Neuroradiology, University Hospital Augsburg, Stenglinstraße 2, 86156 Augsburg, Germany; 2 Department of Diagnostic and Interventional Neuroradiology and Nuclear Medicine, Universitätsklinikum Knappschaftskrankenhaus Bochum, Universitätsklinik der Ruhr-Universität Bochum, Bochum, Germany

**Keywords:** Cerebrovascular disorders, Stroke

## Abstract

The use of stents is well established in the treatment of broad-based intracranial aneurysms. The aim of this study is to report on safety, feasibility and midterm follow-up of the new LVIS EVO braided stent for the treatment of cerebral aneurysms. All consecutive patients with intracranial aneurysms who were treated with the LVIS EVO stent in two high volume neurovascular centers were retrospectively enrolled in this observational study. Clinical and technical complications, angiographic outcome and clinical short-term and midterm results were evaluated. The study included 112 patients with 118 aneurysms. 94 patients presented with incidental aneurysms, 13 patients with acute SAH and 2 patients with acute cranial nerve palsy. For 100 aneurysms a jailing technique was used, re-crossing of the stent was performed in 3 cases. For the residual 15 cases the stent was placed as a bail-out or as a second step. Immediate complete occlusion was observed in 85 aneurysms (72%). Midterm follow-up was available for 84 patients with 86 aneurysms (72.9%). One stent showed asymptomatic complete occlusion on follow-up imaging, in all other cases no in-stent stenosis was observed. The rate of complete occlusion was 79.1% at 6 months and 82.2% at 12–18 months. Midterm follow-up data of this retrospective observational cohort of two neurovascular centers corroborates the safety profile of the LVIS EVO device for treatment of ruptured and unruptured intracranial aneurysms.

## Introduction

The use of stents is well established in the treatment of unruptured intracranial aneurysms, and this technique is increasingly used in the treatment of unruptured aneurysms as well^1,2^. Especially patients with wide-neck aneurysms and bifurcation aneurysms seem to profit from this method^3^. Laser-cut and braided stents are predominantly used for this purpose. Laser-cut stents have been shown to open easily with minimal shortening. Re-crossing is usually possible, so only one microcatheter needs to be used if the stent fits through the same microcatheter as the coils^4^. A disadvantage of these stents is the somewhat poorer confirmation of the stent to the vessel wall, especially with closed-cell designs. This problem is likely to be at least partially addressed with hybrid designs such as a combination of open and closed cells^5^. Braided stents on the other hand show a better wall apposition in curved arteries and provide theoretically a better immediate or progressive occlusion due to a greater metal surface area coverage, although this advantage has not yet been clearly confirmed^5–7^. Another advantage of the small cell size of braided stents compared with laser-cut stents is better protection against protrusion of the coil into the parent vessel during embolization. The LVIS EVO stent (MicroVention, Aliso Viejo, CA, USA) is a relatively new braided self-expanding retrievable microstent that differs from its predecessor in enhanced visibility, smaller cell size and shorter flared ends. Its immediate safety and efficacy has already been demonstrated in small observational cohorts^8–10^. Easy deployment and complete opening even in challenging anatomies like s-shaped curves seems to be a feature of this stent in most cases^8^. The purpose of this study was to provide further data on the periprocedural and midterm safety, feasibility, and efficacy of the LVIS EVO stent in a larger cohort.

## Materials and methods

### Study design

This is a retrospective observational study of two neurovascular institutions. The study was approved by the Institutional Review Board of Ludwig-Maximilians-University Munich (Ethikkommission LMU). Written informed consent was waived by the Ethics Committee that approved this study’s protocol. The study has been performed in accordance with the ethical standards laid down in the 1964 Declaration of Helsinki and its later amendments. All consecutive aneurysms that were treated with the LVIS EVO device between September 2019 and July 2021 were included for further evaluation. Only aneurysms with multiple pretreatments were excluded from the analysis. The decision to use an LVIS EVO stent was at the discretion of the treating interventionalist. Typically, the selection criteria for using the LVIS EVO stent included broad-based aneurysms not suitable for coiling only or balloon-assisted coiling, especially in cases where better visibility of the stent was desirable. It was also used as a bail-out technique in situations of coil protrusion or after using an oversized WEB device. The LVIS EVO stent was preferred over laser-cut stents, particularly in challenging anatomies with extensive curves in the parent vessels, to provide better wall apposition. Data were collected retrospectively from electronic databases. Medical records were screened for several patient parameters including sex, age, clinical presentation, pretreatment mRS score and, in case of acute subarachnoid hemorrhage, Hunt and Hess score, as well. Aneurysms were assessed according to location, type and size. The material used, the interventional procedure, and the peri- as well as postprocedural complications were documented. Finally, data on the clinical and imaging course of the patients and the treated aneurysms were collected.

### Description of the LVIS EVO device

The LVIS EVO stent is a braided self-expanding retrievable microstent. Changes to its predecessor include enhanced visibility, smaller cell size and shorter flared-ends. The LVIS EVO device is compatible with a 0.0165-inch or 0.017-inch microcatheter. Recommended by the manufacturer is the Headway 17 microcatheter (MicroVention) and the double-lumen balloon catheters Scepter C or XC (MicroVention) as delivery systems. Main differences to its predecessors, the LVIS and LVIS Jr. devices (MicroVention), are the compatibility with a smaller microcatheter, shorter flared-ends and the improved visibility due to the drawn filled tube wires, which consist of a nitinol outer material and a platinum core. The stent is re-sheathable and has a smaller cell size than available laser-cut stents with a metal surface area coverage between 17 and 28% depending on vessel anatomy and stent size. Available diameters range between 2.5 and 4 mm.

### Endovascular treatment

All patients without acutely ruptured aneurysm were premedicated with dual antiplatelet therapy (100 mg acetylsalicylic acid (ASA) and 75 mg Clopidogrel daily), starting 5 days prior intervention in elective cases. Responder status was assessed using the Multiplate Test (Roche GmbH, Germany); in case of non- or partial responder status medication was changed accordingly, in most cases from Clopidogrel to Ticagrelor (90 mg twice daily). Acutely ruptured aneurysms were treated intraprocedurally with 100 mg ASA and additionally Heparin or Tirofiban body weight-adapted. Tirofiban was administered intravenously with a dosage of 25 mcg/kg within 3 min during stenting followed by 0.15 mcg/kg/min and continued for up to 24 h. After the procedure, antiaggregation was generally changed to an oral scheme with 100 mg ASA per day and Ticagrelor (90 mg twice a day) or Clopidogrel (75 mg per day). Dual antiplatelet therapy was usually continued for at least 6 weeks after intervention followed by single antiplatelet therapy with 100 mg ASA.

### Patients

**Figure 1:**
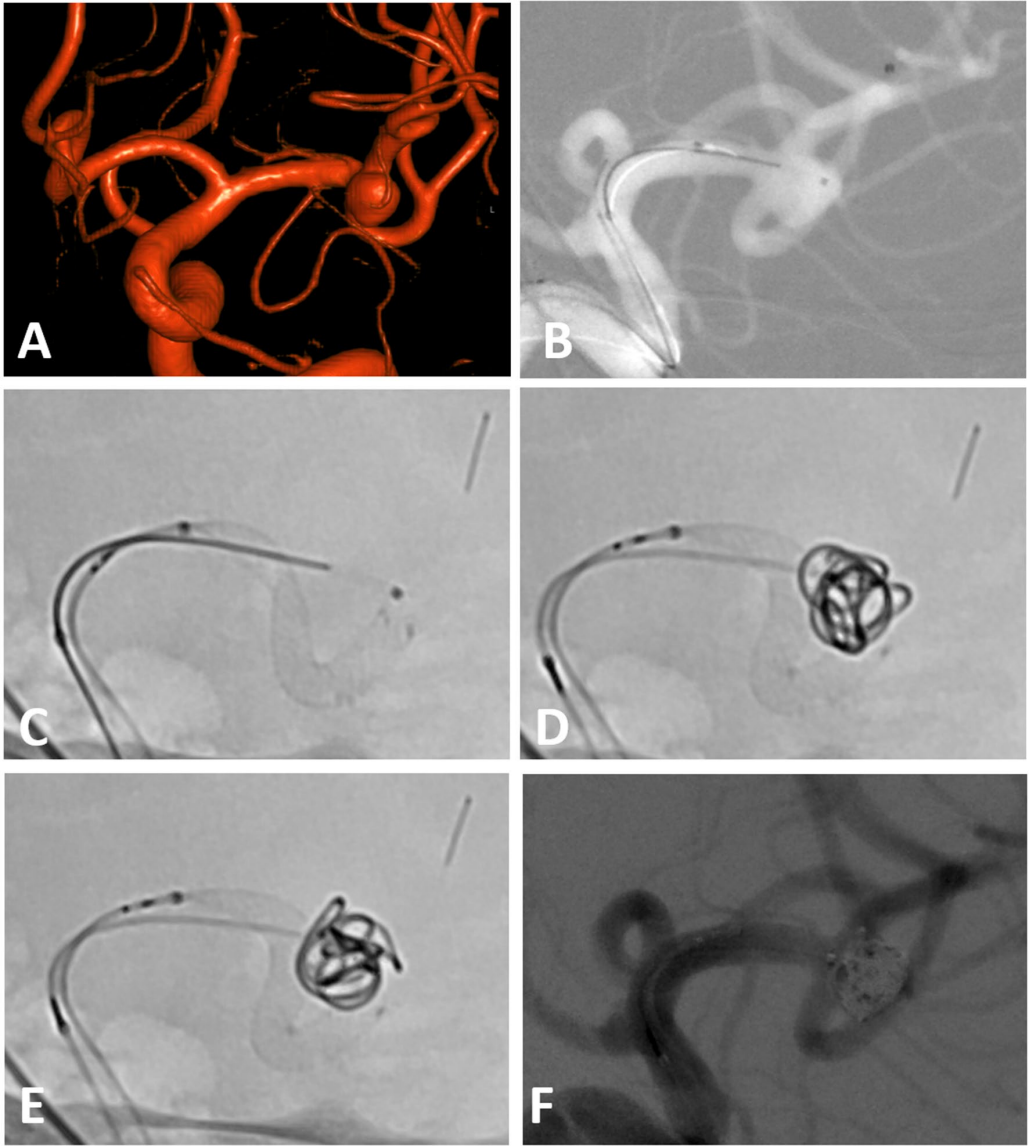
(**A**) Incidental broad-based aneurysm of the left MCA in a female patient in her fifties. (**B**) Jailing technique with one microcatheter within the aneurysm sac and on in the inferior trunk of the MCA .(**C**) First position of the LVIS EVO stent with an incomplete adherence to the vessel wall and a slight kinking—note the very good visibility of the stent wires (**D**) Final position of the stent after partial re-sheathing and re-positioning with formation of a shoulder at the neck. The first coil is already within the aneurysm sac. (**E**) Final position of the framing coil using the repositioned stent as a scaffold (**F**) Final angio run after placement of the last coil.

All procedures were performed using a 6F guiding catheter or an 8F long sheath with or without an intermediate catheter. In the majority of cases a jailing-technique for aneurysm access and coiling was used. Due to the small cell size re-crossing into the aneurysm after stent placement is possible but more difficult than jailing. This necessitates usually the use of two microcatheters during the intervention. After catheterization of the parent vessel and after placing the second microcatheter in the aneurysm, the stent was usually deployed only partially to allow for later repositioning. The better visibility of the stent allows for adapting the stent to challenging anatomies especially at bifurcations. To narrow the neck of a broad-based aneurysm the stent can be deployed using a push-and pull-technique to form a sort of shoulder at the aneurysm neck. This technique may eliminate the need for Y-stenting in some cases and has been described as shelf technique elsewhere^11,12^. An example of this technique is provided in Fig. 1.

### Treatment complications and follow-up

Medication before, during and following treatment, technical procedure and complications were assessed. Occlusion was graded to the Raymond–Roy classification (RROC)^13^. Clinical course until discharge was documented and occlusion grade as well as mRS on follow-up was assessed. Follow-up visits were scheduled usually after 6 months and between 12 and 18 months after treatment according to the standards of the two institutions and depending on patient’s condition, especially after aneurysm rupture. Preferred imaging modality at first follow-up was DSA in combination with MRI and MR-angiography. Further follow-up imaging was generally performed with MRI and MRA. The clinical status of each patient was assessed by a member of the clinical team using the mRS. The impact of the COVID-19 pandemic on postponement of follow-up appointments and choice of follow-up modality was also assessed.

## Results

### Patients

112 patients (median age 58 years; range 38–75 years, 82 female) harboring 118 aneurysms were included in the analysis. 97 aneurysms (82.2%) in 94 patients were discovered incidentally, 13 patients with 15 aneurysms (12.7%) had acute SAH, 2 aneurysms (1.7%) led to acute cranial nerve palsy due to the target aneurysm, 2 patients with 2 aneurysms (1.7%) had acute stroke due to emboli from the target aneurysm, and one patient had an aneurysm (0.8%) symptomatic with seizure. 10 aneurysms (8.5%) were residual aneurysms after endovascular (n = 7; 5.9%) or surgical treatment (n = 3; 2.5%). 107 aneurysms (90.7%) were located in the anterior circulation, 11 (9.3%) in the posterior circulation. The median width of the aneurysm dome was 5 mm (range 1–28 mm), and the median width of the aneurysm neck was 3.9 mm (range 1–19 mm), resulting in a median dome-to-neck ratio of 1.3. All but 3 aneurysms (97.5%) had a dome-to-neck-ratio of ≤ 2. Table 1 summarizes the patient and aneurysm characteristics.

**Table 1:** Patient and Aneurysm characteristics.

	[N]	[%]
Patients	112	100
Female	82	73.2
Male	30	26.8
Aneurysm	118	100
Location		
Middle cerebral artery	44	37.3
Anterior cerebral artery	36	30.5
Internal carotid artery	27	22.9
Basilar artery	6	5.1
Posterior cerebral artery	2	1.7
Posterior inferior cerebellar artery	2	1.7
Vertebral artery	1	0.8

	Median (mm)	Range (mm)
Aneurysm dome	5	1–28
Aneurysm neck	3.9	1–19

### Intervention and angiographic results

A single LVIS EVO stent was used in 107 aneurysms (90.7%). Another device was used in 11 aneurysms (9.3%). The additional device was a Neuroform Atlas Stent (Stryker, Kalamazoo, MI, USA) in 10 cases, implanted with a crossing Y-configuration^14^. Of note, in these 10 cases, the Neuroform Atlas stent was deployed first because of its larger cell size, to avoid the more difficult re-crossing of the LVIS EVO stent with its smaller cell size. In one case, a second LVIS EVO was implanted with a H-configuration for an aneurysm at the anterior communicating artery. In the H-stent configuration, two stents are each inserted on one side from ipsilateral A2 to ipsilateral A1 without crossing the anterior communicating artery, which is usually sacrificed as a result^15^. Coiling of the target aneurysm was performed in 108 cases (92.5%), in 57 cases more than 50% of administered coils were hydrogel coils. A WEB device (MicroVention, Aliso Viejo, CA, USA) was used in 7 cases (5.9%). Stenting in combination with WEB device was used in this context either as a bail-out technique when the WEB was oversized, to push the WEB into the aneurysm, or for unusually complex aneurysms. In one case only a LVIS EVO stent was placed as part of a staged treatment before coiling in a second intervention, in one case, a coil could not be placed in the aneurysms due to the small size of 1.9 mm, so only a LVIS EVO stent was implanted with a pushing technique to condense the cell sizes of the stent and in another case the stent was used for bail-out after the placement and incomplete opening of a flow diverter.

The jailing technique was used in 98 of 108 cases for coiling and in 2 of 7 cases for placing the WEB device. In 3 cases, the stent was re-crossed to gain access to the aneurysm. However, this approach was complicated by the narrow cell size of the stent and led in one case to a re-rupture of an already ruptured aneurysm of the anterior communicating artery. In 15 cases, the stent was deployed either as a second step after coil or WEB device placement or as a bail-out for coil or WEB protrusion in the parent vessel.

Complete occlusion immediately after intervention (RROC 1) was achieved in 85 aneurysms (72.0%), a residual neck (RROC 2) was present in 25 aneurysms (21.2%) and 8 aneurysms (6.8%) showed a residual or complete filling of the aneurysm (RROC 3).

### Intraprocedural complications

In 7 cases angiographic apparent thromboembolic complications were observed, all of which were treated with intravenous Tirofiban or Eptifibatide. In 6 cases the complication resolved without clinical sequelae, in one case a hemiparesis persisted until discharge, resulting in a mRS of 4. Intraprocedural rupture of the target aneurysm occurred in 4 cases, in one of these cases after re-crossing of the stent in a patient with SAH. All intraprocedural ruptures were successfully treated with coiling, two cases without clinical sequelae and in two cases with new minor strokes.

### Early post-procedural complications

Of the 13 patients with acute SAH, 4 developed vasospasms during hospitalization. Three of these patients were treated with intra-arterial spasmolysis, and 2 of the 4 patients died of their vasospasm-induced infarcts during their stay. All vasospasm occurred remotely from the implanted stents and were considered to be SAH-related, not device-related. One patient with SAH had a transient loss of vision after treatment of an aneurysm of the posterior inferior cerebellar artery.

One patient had 4 days after intervention a hemineglect and was treated after exclusion of hemorrhage with intravenous Tirofiban. Ticagrelor was changed to Prasugrel due to assumed non- or partial responder status to Ticagrelor without further symptoms. One patient with an aneurysm of the ophthalmic segment of the ICA suffered a postprocedural occlusion of the central retinal artery which vision loss, which persisted in spite of intraarterial administration of rtPA. The two patients with symptomatic, partially thrombosed aneurysms were also symptomatic in the short-term. The first patient with an aneurysm of the posterior communicating artery showed a new palsy of the third cranial nerve which partially regressed until discharge. The second patient had an acute stent occlusion 8 days after intervention and was treated with thrombectomy. On discharge she still had a left sided hemiparesis with a mRS of 2.

### Late post-procedural complications

A patient with a symptomatic giant aneurysm of the P1 segment of the posterior cerebral artery experienced acute headache with perifocal edema on MRI 49 days after treatment, which was presumably due to acute thrombosis of the aneurysm and was successfully treated with steroids.

One patient presented with a TIA and new infarcts on MRI 2 month after treatment in the territory of the aneurysm at the bifurcation of the middle cerebral artery, which was treated with Y-stenting. DSA showed no in-stent-stenosis. All symptoms resolved completely, ASA was continued for life. No further treatment was required.

In summary treatment with the new LVIS EVO device led to technical complications in 13 patients (11.6%) and to a device related permanent morbidity and mortality in 6 patients (5.3%) and 0 patients (0%), respectively.

### Follow-up results

**Figure 2:**
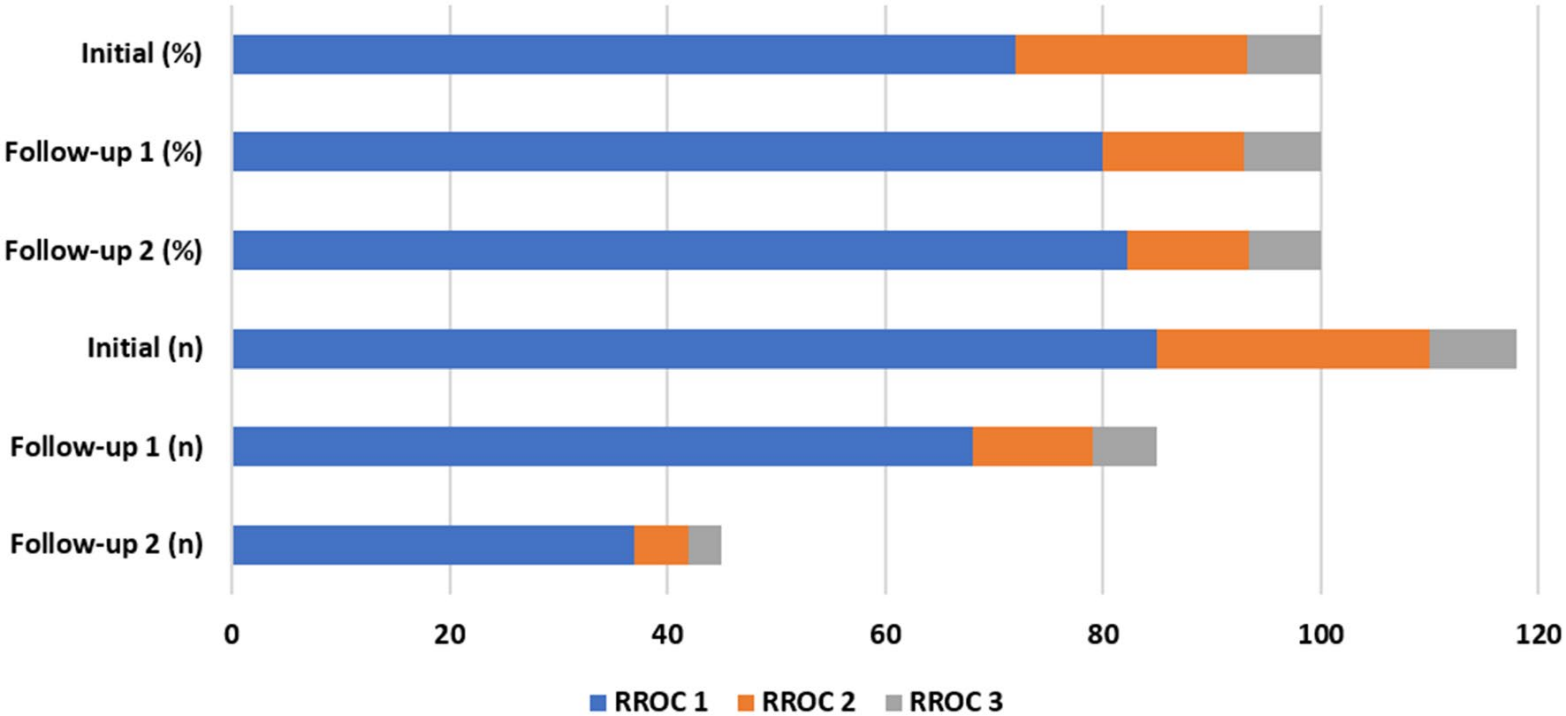
Shift of occlusion rates (percentage and absolute numbers) from treatment to 6 month (Follow-up 1) and 12–18 month follow-up (Follow-up 2).

Follow-up was available for 84 patients with 86 aneurysms (72.9%). Mean time between intervention and last follow-up was 224 days (11–672 days) or about 7.4 months. DSA as follow-up was performed in 62 of these patients (73.8%), the other patients had an MRI with MRA in most cases and in 3 cases a flat detector CT-angiography or conventional CT-angiography, and one patient no vascular imaging at all. Of 26 patients with initially incomplete occlusion 17 progressed to complete occlusion (RROC1) on short-term follow-up (in 12 patients documented by DSA, in 4 patients by MRI with MR-angiography and in one case by CT-angiography), 9 of these patients were treated with hydrogel coils. Another 3 patients progressed to complete occlusion on mid-term follow-up (in 2 patients documented by DSA and in one case by MRI and MR-angiography); one patient progressed from RROC3 to RROC2. Partial recanalization was observed in 7 aneurysms. Occlusion rate and shift in occlusion rate are summarized in Fig. 2 and Table 2.

**Table 2:** Summary of immediate and follow-up results according to the RROC; FU1 = Follow-up after 6 months, FU2 = Follow-up after 12–18 month.

	Immediate N [%]	FU1 N [%]	Progress to RROC1 on FU1 N [%]	FU2 N [%]	Progress to RROC1 on FU2 N [%]
Aneurysms	118	85	17 [19.8%]	45	3 [6.7%]
RROC1	85 [72.0]	68 [79.1]		37 [82.2]	
RROC2	25 [21.2]	11 [12.8]		5 [11.1]	
RROC3	8 [6.8]	6 [7.0]		3 [6.7]	

3 patients were re-treated due to residual aneurysms between 6 and 8 months after the first intervention. One patient had an asymptomatic stent occlusion. Beyond that, no in-stent-stenoses were observed on follow-up imaging. Procedural complications and outcome are summarized in Table 3.

**Table 3:** Complications and outcome.

	Patients N [%]
Procedural complications	13 [11.6]
Hemorrhagic complications	4 [3.6]
Ischemic complications	7 [6.3]
Mortality at discharge	2 [1.8]
Favorable outcome at discharge (mRS ≤ 2)	106 [94.6]
Re-treatment rate	3 [2.7]

The impact of the COVID-19 pandemic on follow-up was small. In 3 cases, intravenous flat detector CT angiography was performed rather than DSA because of restrictions in patient admission. In 4 cases, follow-up appointments were postponed. For these patients instead of 6 months after treatment, follow-up visits were performed between 7.5 and 17 months after the procedure.

## Discussion

This cohort is to our knowledge the largest cohort of patients treated with the LVIS EVO device to date. Our results confirm the safety and efficacy of the new LVIS EVO device in the treatment of ruptured and unruptured intracranial aneurysms with an adequate occlusion rate on midterm follow-up despite a rather inhomogeneous patient cohort. There are already several case series with experiences on the LVIS EVO device, reporting early experiences, though. Sirakov et al. describe their first six patients treated with LVIS EVO with a 100% success rate and a 100% immediate occlusion rate without device-related complications^16^. Poncyljusz and Kubiak report 30 patients with 35 aneurysms with again a 100% success rate, a 100% initial occlusion rate and one thromboembolic complication^9^. Vollherbst et al. describe in 57 patients with 59 aneurysms a complete initial occlusion rate of 54.2% and periprocedural complications in 11.9%, mostly thrombus-formation^8^. Several patients from the series of Vollherbst are also part of our current cohort. And finally, a multicenter retrospective study reports on 15 patients treated with LVIS EVO. They found complete occlusion after the procedure in 66.7% and thromboembolic complications in these cases^10^.

LVIS EVO complements the range of braided stents on the market. However, clear differentiation criteria for the selection of the stent type do not yet exist. On the one hand, laser-cut stents are known to open more easily without relevant shortening but inferior conformation to the vessel wall. Braided stents on the other side have a theoretical advantage over laser-cut stents because of their small cell size and the ability to further reduce cell size by condensing the stent with moderate pressure, especially across the aneurysm neck. Theoretically, they may have a flow-diverting effect, leading to less frequent recanalization and possibly to progressive occlusion of an incompletely occluded aneurysm at follow-up^17,18^. This effect may explain the progressive occlusion of 20 patients on follow-up imaging in our cohort. In one of our cases, in which coils could not be placed because of the small size of the aneurysm and unfavorable anatomy, the stent was effectively compacted over the aneurysm neck. This aneurysm showed complete occlusion on follow-up imaging after 6 months as a possible correlate of a flow diverter-like effect, as it has been described for other braided stents^19,20^. In contrast to most flow diverters, the stent can be used with a 0.0165-inch or 0.017-inch microcatheter and can be applied to bifurcation aneurysms, such as those at the bifurcation of the middle cerebral artery, without hemodynamically compromising the second branch. However, while re-crossing the stent is an option if necessary, it is not recommended for standard use. Compared with data of laser-cut stents from the same two centers^4^, improved occlusion was diagnosed in 19.8% with LVIS EVO compared with 7.7% with Neuroform Atlas. However, the extent to which the increased use of hydrogel coils in the current cohort influenced the superior 6-month occlusion rate remains unclear.

The LVIS and LVIS Jr. stents are well-established stents for stent-assisted coiling and are predecessors to the LVIS EVO device^21^. The LVIS EVO, however, has the advantage of being compatible with a smaller microcatheter, which enables easier and safer navigation to more distal locations, even in tortuous anatomies. Moreover, the shorter flared ends of the LVIS EVO correspond with a larger working length compared to LVIS and LVIS Jr. stents of the same total length, allowing for greater variability when placing the stent, particularly in challenging anatomies. Finally, the LVIS EVO offers better visibility, which is a crucial feature, particularly at the level of the skull base with its overlying bone structures.The disadvantage of the smaller cell size of the LVIS EVO is that re-crossing the stent is more difficult than using a jailing technique. This is mirrored in our patient who experienced an aneurysm re-rupture with progressive SAH after a re-crossing attempt, so jailing is the recommended approach if using the LVIS EVO device contrary to the preferred approach of re-crossing for laser-cut stents^4^. The same applies if crossing Y-stenting is an option. Due to the small cell size of the LVIS EVO, a stent with a larger cell size must be selected as the first stent. However, the so-called shelf technique may eliminate the need for Y stenting in some cases^11,12,22^. This technique can be applied with the LVIS EVO due to the enhanced visibility of all stent wires and the resheathability and might be especially helpful in aneurysms of the middle cerebral artery^23,24^. However, clear discriminatory criteria for the use of braided stents versus laser-cut stents cannot be established on the basis of our data. Further studies are needed in this regard.

The management of partially thrombosed aneurysms remains controversial^25,26^. In our series two patients with partially thrombosed aneurysms were treated with stent assisted coiling (SAC). Both patients were symptomatic after the procedure, one with third nerve palsy and the other with a stent occlusion treated with thrombectomy 8 days after intervention. Although smaller cell size might be beneficial for preventing arterio-arterial emboli originating from the aneurysm, other factors such as induction of thrombi within the stent or the parent vessel may prevail. Therefore, the ideal choice of stent for this type of aneurysm remains unclear.

In our series technical complications occurred in 11.6% of patients and a device related permanent morbidity in 5.3%. Immediate occlusion was observed in 72.0% of all aneurysms which proceeded to complete occlusion in additional 20 patients on follow-up. These data are in line with published data on SAC in general and with different devices^6,21,27–29^. However, our data allow no direct comparison with other devices regarding efficacy or individual preference depending on aneurysm type, size and features.

### Limitations

Our data represent a retrospective observational cohort of only two-centers with self-assessment of angiographic outcome and complications and without a control group. Furthermore, the modality of follow-up imaging was DSA in just under three-quarters of cases. This leads to obvious limitations in interpreting and generalizing our results. However, we can add a comparatively large cohort with midterm results to the growing data on LVIS EVO and aneurysm treatment with braided stents. Another limitation is the inhomogeneous patient cohort, as we included different aneurysm types in a wide range of locations and sizes since we included almost all consecutive cases in our centers treated with LVIS EVO. On the other hand, this demonstrates the applicability of the device not only in selected case but in a broad spectrum of aneurysms. Furthermore, patients were partially treated and scheduled for follow-up during the ongoing COVID-19 pandemic, however the impact on choice of modality and follow-up date was small. And finally, there is no long-term follow-up yet, as the stent was introduced relatively recently.

## Conclusion

Short- and midterm follow-up data of this retrospective observational cohort of two neurovascular centers corroborates the safety profile of the LVIS EVO device for treatment of ruptured and unruptured intracranial aneurysms. LVIS EVO in combination with hydrogel coils may support progressive occlusion on short-term follow-up.

## Data Availability

Data supporting this study’s findings are available within the article or from the corresponding author upon reasonable request.
